# The importance of phenotypic data analysis for genomic prediction - a case study comparing different spatial models in rye

**DOI:** 10.1186/1471-2164-15-646

**Published:** 2014-08-04

**Authors:** Angela-Maria Bernal-Vasquez, Jens Möhring, Malthe Schmidt, Manfred Schönleben, Chris-Carolin Schön, Hans-Peter Piepho

**Affiliations:** Bioinformatics Unit, Institute of Crop Science, University of Hohenheim, Fruwirthstrasse 23, 70599 Stuttgart, Germany; KWS LOCHOW GMBH, Ferdinand-von-Lochow-Strasse 5, 29303 Bergen, Germany; Plant Breeding, Technische Universität München, Liesel-Beckmann-Strasse 2, 85354 Freising, Germany

**Keywords:** Stage-wise analysis, Genomic prediction, Cross validation, Spatial models, Multi-environment trials (MET), Restricted maximum likelihood (REML)

## Abstract

**Background:**

Genomic prediction is becoming a daily tool for plant breeders. It makes use of genotypic information to make predictions used for selection decisions. The accuracy of the predictions depends on the number of genotypes used in the calibration; hence, there is a need of combining data across years. A proper phenotypic analysis is a crucial prerequisite for accurate calibration of genomic prediction procedures. We compared stage-wise approaches to analyse a real dataset of a multi-environment trial (MET) in rye, which was connected between years only through one check, and used different spatial models to obtain better estimates, and thus, improved predictive abilities for genomic prediction. The aims of this study were to assess the advantage of using spatial models for the predictive abilities of genomic prediction, to identify suitable procedures to analyse a MET weakly connected across years using different stage-wise approaches, and to explore genomic prediction as a tool for selection of models for phenotypic data analysis.

**Results:**

Using complex spatial models did not significantly improve the predictive ability of genomic prediction, but using row and column effects yielded the highest predictive abilities of all models. In the case of MET poorly connected between years, analysing each year separately and fitting year as a fixed effect in the genomic prediction stage yielded the most realistic predictive abilities. Predictive abilities can also be used to select models for phenotypic data analysis. The trend of the predictive abilities was not the same as the traditionally used Akaike information criterion, but favoured in the end the same models.

**Conclusions:**

Making predictions using weakly linked datasets is of utmost interest for plant breeders. We provide an example with suggestions on how to handle such cases. Rather than relying on checks we show how to use year means across all entries for integrating data across years. It is further shown that fitting of row and column effects captures most of the heterogeneity in the field trials analysed.

**Electronic supplementary material:**

The online version of this article (doi:10.1186/1471-2164-15-646) contains supplementary material, which is available to authorized users.

## Background

Genomic prediction (GP) was first introduced in 2001 [1] as a method that allows the prediction of genomic estimated breeding values (GEBV) for plants and animals by using information of genetic markers. In plant breeding, GP has been adopted as another stage of the breeding scheme [2], not diminishing the importance of the phenotypic analysis usually carried out in several environments. Merging the phenotype and the genotype analyses has been addressed through the so-called stage-wise analysis [3]. In the first stage environments are analysed separately and genotype means are computed and then submitted in the GP stage to predict GEBV based on dense genetic markers such as single nucleotide polymorphisms (SNPs).

In plant breeding, assessing genotypic adaptability and stability, and predicting breeding values of the genotypes in other environments and other years, makes use of multi-environment trials (METs), which aim to evaluate as many genotypes as possible in as many as possible locations [4–7]. These METs are typically laid out as generalised lattice designs testing a large number of different genotypes per trial. The number of tested genotypes is limited by factors such as seed production, production cycle length and availability of physical resources, e.g. land and budget [8].

Within years, genotypes are tested in series of trials, which are connected by checks. Checks are lines grown in every trial as controls because their performance is known and/or they are already commercial material. Checks can be also used to connect years. In the rye breeding program considered in this paper, a completely different set of genotypes is tested in each year, but these genotypes are from the same breeding population. The accuracy of a genomic prediction model depends on the number of genotypes used for calibration. So there is definitely a need to combine data across years. Low connectivity across years is a challenge when trying to combine data across years, and this is one main motivation for this paper. Furthermore, the unbalancedness due to the design layout and the different and large number of evaluated genotypes increases the heterogeneity introducing high complexity to the variance-covariance structure among adjusted genotype means [3].

Analysis of METs could be done as single-stage analysis, modelling the complete observed data at the level of individual plots, or using a stage-wise approach, where experiments are analysed first at the level of environments (or trials), obtaining adjusted means per genotype, which are then summarised across environments (or trials) in the next stage [3]. A single-stage analysis accounts entirely for the variance-covariance structure of the recorded observations [6], therefore it is regarded as the gold standard. However, it has been shown that in a stage-wise analysis, a loss of information occurring in the transition through stages can be minimized by an appropriate weighting scheme [9].

If feasible, a single-stage approach is preferable to a stage-wise analysis [10]. Nevertheless, the latter is acceptable for GP, since it is simple, computationally more efficient and also allows to easily account for any specifics of randomisation layout and error modelling for each environment [3]. It should be stressed, however, that in a stage-wise analysis the weights are chosen to approximate the variance-covariance matrix of adjusted means from previous stages. We used here a three-stage approach and compared different spatial correlation structures in the first stage to correct field heterogeneity at the trial level.

Spatial error models may provide more accurate estimates of genotype effects than models not accounting for spatial adjustment [11, 12] but they are computationally more demanding and convergence may be difficult to reach. Any effort in terms of improving the genomic predictions would include checking if these improved estimates have an effect on the predictive ability when markers are added to the model. The performance of alternative spatial models can be assessed by *k*-fold cross validation (CV).

Similarly, the merits of different spatial models used to compute adjusted means in the first stage can be compared by the same CV procedure, if the same GP procedure is used for each analysis. This suggests that genomic prediction-cross validation (GP-CV) can be used to identify the best-fitting mixed model in stage one. The common method of model selection makes use of information criteria based on the log likelihood, e.g. the Akaike information criterion (AIC) or the Bayesian information criterion (BIC) [13]. When the restricted maximum likelihood (REML) method is used, models can only be compared by information criteria if they have the same fixed effects; otherwise, the maximum likelihood (ML) method should be used [13]. CV is, in this sense, not used to tune parameters as in many penalization methods (e.g. adaptive Lasso, SCAD (Smoothly Clipped Absolute Deviation), machine learning methods) but only as a tool to compare models that use REML. REML is considered the best available method of variance parameter estimation, preferable to ML [14]. Consequently, it is of interest to devise model selection procedures that can use REML and also can compare models with different fixed effects. GP-CV has already been used to judge environments in order to optimise the accuracy in GP [15]. We used this tool here as model selection method in comparison to the traditional use of AIC.

The aims of this work were: i) to assess the advantage for the predictive ability when using a spatial model for phenotypic analysis, ii) to compare stage-wise approaches for GP when the data are weakly connected across years, and iii) to compare AIC and GP-CV as methods of selection of models for phenotypic data analysis towards GP in rye.

## Methods

### Field layout and data set

A commercial rye breeding program by KWS-LOCHOW established in Poland and Germany aims to develop superior hybrid varieties for the seed market. The implementation of GP within the breeding program makes use of the measurements of hybrid performance of the first cycles of phenotypic evaluation of the material (Cycle1). Selections made in Cycle1 are intensively evaluated in further cycles, aiming to double-check the selection decisions. For our purposes, these additional cycles do not add much useful information. Hence, we used only the first cycles of the program. The populations tested in each year consist of S _2_ genotypes, which display genetic relatedness and population stratification due to complex genealogical history [16].

Besides the phenotypic data, a 16K Infinium iSelect HD Custom BeadChip was used to characterise 1610 individuals from Cycle1-2009 and Cycle1-2010 and 6 checks. Several traits were evaluated during this project: grain dry matter yield, plant height and thousand kernel weight, as well as ordinal scores of rust, mildew and lodging among others. In this work we used grain dry matter yield measurements of the phases of selection Cycle1-2009, Cycle1-2010 and Cycle1-2012, and marker information for the genotypes of 2009 and 2010. Although no marker information of year 2012 was available, it makes sense to use this dataset to observe the trend in one additional year and in this way, support the results of the phenotypic analysis of previous years.

A Cycle1 experiment consists of subsets of 320 genotypes from the S _2_ populations tested in several locations within each of the two countries involving two testers (Tables 1 and 2). We define a trial as the physical unit within a location, where a subset of genotypes that were testcrossed to the same tester is evaluated. Trials at a location were laid out as *α*-designs with two replicates. Each trial was randomized independently from the others using the software CycDesign (VSN International; http://www.vsni.co.uk/). (However, we are aware that some breeders tend to use the same randomization layout in several locations. Ideally, each trial should have a different randomization). In our notation, trials of a Cycle1 experiment are labelled as S1, S2, …, S24. Row and column coordinates of the plots to account for spatial variation are available.

**Table 1:** **General representation of the testers by locations (Loc) by years classification of Cycle1 year 2009 and 2010 in Germany (G-L1, ⋯, G-L8) and Poland (P-L1, ⋯, P-L4)**

Loc	Cycle1-2009						Cycle1-2010					
	Tester1			Tester2			Tester3			Tester4		
G-L1	S1	S2	S3				S10	S11	S12			
G-L2	S1	S2	S3					S11		S10		
G-L3	S1	S2	S3									
G-L4	S1	S2	S3	S1	S2	S3	S10	S11	S12	S10	S11	S12
G-L5				S1	S2	S3				S10	S11	S12
G-L6				S1	S2	S3				S10	S11	S12
G-L7				S1	S2	S3					S11	S12
G-L8							S10	S11	S12			
P-L1	S7	S8	S9	S7	S8	S9	S13	S14	S15	S13	S14	S15
P-L2	S7	S8	S9	S7	S8	S9	S13	S14	S15	S13	S14	S15
P-L3	S7	S8	S9	S7	S8	S9	S13	S14	S15	S13	S14	S15
P-L4	S7	S8	S9	S7	S8	S9	S13	S14	S15	S13	S14	S15

Series of trials are represented with the labels S1, S2, ⋯ S15.

**Table 2:** **General representation of the testers by locations (Loc) classification of Cycle1 year 2012 in Germany (G-L4, ⋯, G-L11) and Poland (P-L1, ⋯, P-L6)**

Loc	Cycle1-2012													
		Tester5							Tester6					
G-L4		S16		S17		S18								
G-L5									S16		S17		S18	
G-L6									S16		S17		S18	
G-L7									S16		S17		S18	
G-L8				S17		S18								
G-L9		S16		S17		S18								
G-L10		S16							S16		S17		S18	
G-L11		S16		S17		S18								
P-L1		S19	S20	S21	S22	S23	S24		S19		S21		S23	
P-L2		S19	S20	S21	S22	S23	S24		S19	S20	S21	S22	S23	S24
P-L3		S19	S20	S21	S22	S23	S24		S19	S20	S21	S22	S23	S24
P-L4			S20		S22		S24		S19	S20	S21	S22	S23	S24
P-L5		S31		S33		S35								
P-L6										S20		S22		S24

Series of trials are represented with the labels S16, S17, ⋯ S24.

Normally throughout the program, only a single tester was used per location and year, but in some locations, some subsets of genotypes were testcrossed with the two available testers. This is the case, for example, for location G-L4 in Cycle1-2009, where the genotypes evaluated in the trials S1, S2 and S3 were testcrossed with both Tester1 and Tester2, and it is also the case of locations P-L1, P-L2, P-L3 and P-L4 evaluating genotypes of trials S7, S8 and S9 with both testers. In each year, four common checks were testcrossed with the testers and grown twice in each trial. Over the years 2009 and 2010 one check was in common and none was shared with 2012 (Table 3).

**Table 3:** **Year x Check classification in Germany (G) and Poland (P)**

	2009		2010		2012	
	G	P	G	P	G	P
Check1	x	x				
Check2	x	x				
Check3	x	x	x	x		
Check4	x	x				
Check5			x	x		
Check6			x	x		
Check7			x	x		
Check8					x	x
Check9					x	x
Check10					x	x
Check11					x	x
Check12					x	x
Check13						x

The field layout of some trials was not perfectly rectangular. Some trials at a given location and year had fewer blocks but larger size, i.e., there were two different block sizes within a few trials. Blocks were nested within rows of the field layout.

In the genetic dataset, homozygous marker genotypes were coded as -1 and 1, and the heterozygous type, missing values and technical failures were coded as 0. 58.7*%* of the markers corresponded to homozygous alleles and 16.1*%* were heterozygous. Only a 0.03*%* of the markers were recorded as missing values or technical failures; therefore, an imputation method would not have a strong impact on the subsequent analyses. Monomorphic markers and markers with minor allele frequency (MAF) less than 1% or missing information of more than 10% per marker were dropped. A total of 11285 markers passed the quality test and were used for GP.

### Models

**Figure 1:**
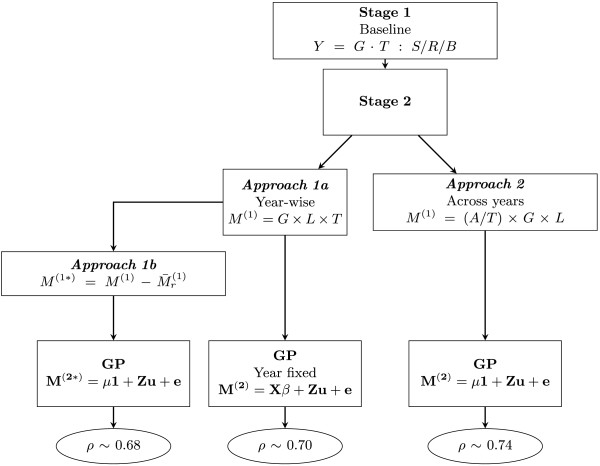
**General representation of stage-wise approaches to compare year-effect adjustment.** Factors were genotype (*G*), tester (*T*), location (*L*), year (*A*), trial (*S*), replicate (*R*) and block (*B*). Grain dry matter yield (*Y*) is the response variable in the first stage, *M* ^(1)^ is the adjusted mean of genotypes across locations used in the second stage, *M* ^(1∗)^ is the year effect-corrected genotype adjusted mean,  represents the simple mean of genotypes of the *r*-th year. In the genomic prediction (GP) stage, *M* ^(2)^ is the *n*×1 vector of adjusted means of genotypes by year for *Approach 1a* and across years for *Approach 2*, *M* ^(2∗)^ is the *n*×1 vector of adjusted means of year effect-corrected genotypes in *Approach 1b*, **X** and **β** are respectively the design matrix and parameter vector of fixed effects, **Z** is the *n*×*p* marker matrix, **u** is the *p*-dimensional vector of SNP effects and **e** the error vector. *Y*=*G*·*T*:*S*/*R*/*B* is the shorthand notation of the model eq. (1) in the text: *Y*_*hijkv*_=(*G* *T*)_*hv*_+*S*_*i*_+*R*_*ij*_+*B*_*ijk*_+*e*_*hijkv*_, *M* ^(1)^=*G*×*L*×*T* stands for the model eq. (2) in the text: , and *M* ^(1)^=(*A*/*T*)×*G*×*L* represents the extended model eq. (4) in the text: . The final predictive abilities (*ρ*) are presented in the ellipses.

**Figure 2:**
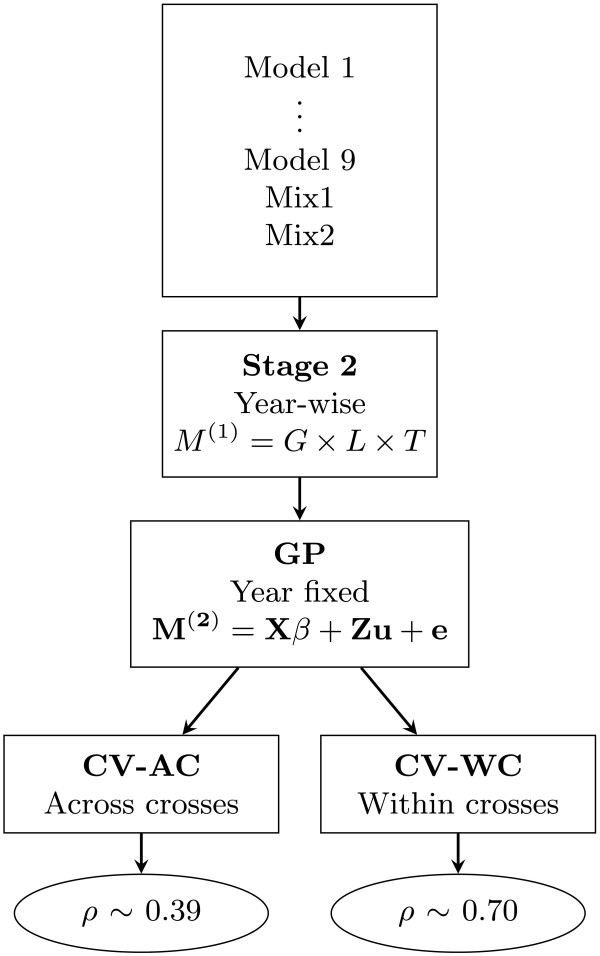
**General representation of model comparison through all the stages of the analysis.** Datasets generated from 9 spatial and non-spatial models plus two mixed datasets generated from best models given the Akaike information criterion (Mix1) and the predictive abilities (Mix2). Factors in second stage were genotype (*G*), location (*L*) and tester (*T*). *M* ^(1)^ represents the adjusted mean of genotypes across locations and years. *M* ^(1)^=*G*×*L*×*T* is the shorthand notation for . In the genomic prediction (GP) stage *M* ^(2)^ is the adjusted mean of genotypes across locations, **X** and **β** are respectively the design matrix and parameter vector of fixed effects, **Z** is the *n*×*p* marker matrix, **u** is the *p*-dimensional vector of SNP effects and **e** the error vector. Sampling methods in cross validation (CV) were across crosses (AC) and within crosses (WC). The final predictive abilities (*ρ*) are presented in the ellipses.

In this section we present the models used in the first stage of the analysis and the models of the approaches followed to adjust the year effect either in the second or the third stage. Figures 1 and 2 depict a general scheme that helps visualizing the methodology.

#### First stage

In the first stage we computed adjusted genotype means by location and year. The factors used for the analysis were genotypes (*G*), testers (*T*), trials (*S*), replicates (*R*) nested within trials and blocks (*B*) nested within replicates. We defined a baseline model as
1

where *Y*_*hijkv*_ is the observed grain dry matter yield of the *h*-th genotype testcrossed with the *v*-th tester in the *k*-th block within the *j*-th replicate of the *i*-th trial, (*GT*)_*hv*_ is the effect of the *h*-th genotype testcrossed with the *v*-th tester, *S*_*i*_ is the effect of the *i*-th trial , *R*_*ij*_ is the effect of the *j*-th replicate nested within the *i*-th trial , *B*_*ijk*_ is the effect of the *k*-th block nested within the *j*-th replicate of the *i*-th trial  and *e*_*hijkv*_ is the plot error associated with the *Y*_*hijkv*_ observation . In model equation (1) we assumed genotypes crossed with testers as a fixed effect to be able to compute genotype adjusted means per tester, whereas the other effects were considered as random effects due to the nested design structure [17].

Table 4 summarises the further models. Some SAS code to fit the first stage models is provided in the supplementary material (Additional file 1). The first model (M1) will be referred to as the baseline model because it was the simplest model and represented the randomisation structure. In the second model (M2) we considered additionally the effects of the *o*-th row (*W*_*ijo*_) and the *q*-th column (*V*_*ijq*_) both within the *j*-th replicate of the *i*-th trial. Subsequently, we added a spatially correlated residual plot effect different from the baseline model, which uses the independent model (ID) with homogeneous variances. We fitted one- and two-dimensional spatial models with and without the so-called nugget, a geostatistical term to designate an independent error effect. As one-dimensional models we used the autoregressive AR(1) variance-covariance nested within blocks without nugget (M3) and with nugget (M7), and linear variance LV within blocks with nugget (M4). In the AR(1) we accounted for the correlation between plots in the same block assuming an exponential decay of correlation with distance, whereas by using LV, it is assumed that the covariance among plots in the same block decays linearly with spatial distance [18, 19]. The most common extension of the spatial model in two dimensions is the direct product structure AR(1) × AR(1), which assumes that an AR(1) model holds both along rows and along columns [20]. The twodimensional models were fitted along rows and columns within replicates without nugget (M5), with nugget (M8), adding rows and columns as effects without nugget (M6) and with nugget (M9). The LV model can also be extended in two dimensions [21]; however, for METs, where the arrangement of the plots might not be perfectly rectangular, this LV × LV model was cumbersome to fit with the software we used, thus we did not consider this model.

**Table 4:** **Spatial and non-spatial models used for the first stage**

Label	Model	Variance-covariance
		structure for error
M1	*Y*_*hijkv*_=(*G* *T*)_*hv*_+*S*_*i*_+*R*_*ij*_+*B*_*ijk*_+*e*_*hijkv*_	ID
M2	*Y*_*hijkoqv*_=(*G* *T*)_*hv*_+*S*_*i*_+*R*_*ij*_+*B*_*ijk*_	ID
	+*W*_*ijo*_+*V*_*ijq*_+*e*_*hijkoqv*_	
M3	*Y*_*hijkv*_=(*G* *T*)_*hv*_+*S*_*i*_+*R*_*ij*_+*B*_*ijk*_+*e*_*hijkv*_	AR(1) within *B*
M4	*Y*_*hijkv*_=(*G* *T*)_*hv*_+*S*_*i*_+*R*_*ij*_+*B*_*ijk*_+*e*_*hijkv*_	LV within *B* + nugget
M5	*Y*_*hijkoqv*_=(*G* *T*)_*hv*_+*S*_*i*_+*R*_*ij*_+*B*_*ijk*_	AR(1) × AR(1) within *R*
	+*W*_*ijo*_+*V*_*ijq*_+*e*_*hijkoqv*_	
M6	*Y*_*hijkv*_=(*G* *T*)_*hv*_+*S*_*i*_+*R*_*ij*_+*B*_*ijk*_+*e*_*hijkv*_	AR(1) × AR(1) within *R*
M7	*Y*_*hijkv*_=(*G* *T*)_*hv*_+*S*_*i*_+*R*_*ij*_+*B*_*ijk*_+*e*_*hijkv*_	Model 3 + nugget
M8	*Y*_*hijkv*_=(*G* *T*)_*hv*_+*S*_*i*_+*R*_*ij*_+*B*_*ijk*_+*e*_*hijkv*_	Model 5 + nugget
M9	*Y*_*hijkoqv*_=(*G* *T*)_*hv*_+*S*_*i*_+*R*_*ij*_+*B*_*ijk*_	Model 6 + nugget
	+*W*_*ijo*_+*V*_*ijq*_+*e*_*hijkoqv*_	

*Y*_*hijkv*_ is the observed dry matter yield of the *h*-th genotype testcrossed with the *v*-th tester in the *k*-th block within the *j*-th replicate of the *i*-th trial, (*G* *T*)_*hv*_ is the effect of the *h*-th genotype testcrossed with the *v*-th tester, *S*_*i*_ is the effect of the *i*-th trial , *R*_*ij*_ is the effect of the *j*-th replicate nested within the *i*-th trial , *B*_*ijk*_ is the effect of the *k*-th block nested within the *j*-th replicate of the *i*-th trial  and *e*_*hijkv*_ is the plot error associated with the *Y*_*hijkv*_ observation . In the models including row and column effects, *W*_*ijo*_ is the effect of the *o*-th row within the *j*-th replicate of the *i*-th trial  and *V*_*ijq*_ is the effect of the *q*-th column within the *j*-th replicate of the *i*-th trial . Spatial variance-covariance structure were independent (ID), autoregressive in one direction [AR(1)], one-dimension linear variance (LV) and two-dimension autoregressive [AR(1) × AR(1)].

Note that we use (*GT*)_*hv*_ as fixed effect, which is necessary to obtain the genotype by tester means. The purpose is also to recover the information of the entries that are grown in the same locations but using different testers (e.g. in Cycle1 location G-L4 and the Polish locations P-L1 to P-L4), so that we captured the effect of the tester in the shared locations.

#### Second stage

In the second stage we computed genotype means across locations and testers. This was done either separately for each year (*Approach 1*) or also averaging across years (*Approach 2*). The years 2009 and 2010, where molecular marker data were available, were connected through only one check. The resulting fundamental question is then how to fit the year effect. Either the year effect is estimated by the mean of all tested entries *(Approach 1)* or we rely on the adjustment by the one single check *(Approach 2)*. We assume that genotypes tested in each year can be regarded as a random sample from the same parent population. Based on the structure of the breeding program, this is a realistic assumption that motivates the approaches described in the following.

Both approaches were compared using the *M*^(1)^ resulting from the analysis of the baseline model in the first stage.

#### Approach 1: Year-wise analysis

Each year was analysed in the second stage using a three-way interaction model of genotypes (*G*), locations (*L*) and testers (*T*) as factors to obtain adjusted genotypes means of each year. The model was
2

where  represents the adjusted mean of grain dry matter yield of the *h*-th genotype, testcrossed with the *v*-th tester in the *s*-th location, *G*_*h*_, *L*_*s*_ and *T*_*v*_ are the main effects of the *h*-th genotype, the *s*-th location and the *v*-th tester, respectively, (*GL*)_*hs*_, (*GT*)_*hv*_ and (*LT*)_*sv*_ are the two-way interaction effects, (*GLT*)_*hsv*_ is the effect of the three-way interaction and *e*_*hsv*_ is the residual error associated with , with  the variance of the *hsv*-th adjusted mean  obtained in the first stage.

Location was considered as random effect  and hence, all the interactions containing this factor are random [17]. The crossed effect of genotypes and testers [ (*GT*)_*hv*_] could have been a fixed effect since genotypes and testers are taken as fixed factors in this stage. However, the crossed effects that include *G* were taken as random here because the factor genotype was used as random in the GP stage. But note that in the first and the second stage we needed to take genotype main effects as fixed in order to compute adjusted means [3]. Besides, since not every genotype was tested with every tester (e.g. in Cycle1 locations G-L1 to G-L3 and G-L5 to G-L8), we needed to take (*GT*)_*hv*_ random to be able to estimate genotype means across levels of testers.

In this approach, the year effect was adjusted in two ways, hereafter referred as to *Approach 1a* and *Approach 1b*. *Approach 1a* used years as fixed factors in the GP stage and *Approach 1b* used a manual adjustment after the second stage by simply calculating the mean of the genotypes by year  and subtracting it to each genotype adjusted mean of the corresponding year (Figure 1). The rationale behind the latter approach is the assumption that the correction for the year effect is better represented by the simple mean of the complete sample of genotypes per year than by just a few checks. The resulting year effect-corrected genotype means  are forwarded to the GP stage, and through CV are evaluated as predictors.

As in the transition from the first to the second stage, there is a loss of information in passing on from the second to the third stage because the (*GLT*)_*hsv*_ effect is confounded with the residual error term. This loss can be minimized by weighting the adjusted means [3]. We used the Smith et al. scheme [6], where adjusted means are weighted by the diagonal elements of the inverse of their variance-covariance matrix computed in the first stage.

At this stage, we computed the heritability for each year using the *ad hoc* method described in Piepho and Möhring [22] as
3

where  is the genetic variance and  is the mean variance of a difference of two adjusted genotype means, corresponding to the best linear unbiased estimators (BLUE). Even though this is not the best method to estimate heritability [23], the square root of this heritability estimate gives a rough idea of an upper limit for the predictive abilities.

#### Approach 2: Across years analysis

The model to account for the year effect in the second stage through the shared check was
4

where  represents the adjusted mean of grain dry matter yield of the *h*-th genotype, testcrossed with the *v*-th tester, in the *s*-th location and *r*-th year, *G*_*h*_ is the main effect of the *h*-th genotype, *L*_*s*_ is the main effect of the *s*-th location and *D*_*rv*_ the main effect of the *v*-th tester within the *r*-th year, which can be extended as *D*_*rv*_=*A*_*r*_+(*AT*)_*rv*_, with *A*_*r*_ the effect of the year and *T* denoting the tester [17]. (*GD*)_*hrv*_, (*GL*)_*hs*_ and (*LD*)_*rsv*_ are the two-way interaction effects, (*GLD*)_*hrsv*_ is the effect of the three-way interaction and *e*_*hrsv*_ is the residual error associated to , with  the variance of the *hrsv*-th adjusted mean  obtained in the first stage. The effects containing *D*_*rv*_ can be extended as (*GD*)_*hrv*_=(*GA*)_*hr*_+(*GAT*)_*hrv*_, (*LD*)_*rsv*_=(*LA*)_*rs*_+(*LAT*)_*rsv*_ and (*GLD*)_*hrsv*_=(*GLA*)_*hrs*_+(*GLAT*)_*hrsv*_.

We considered genotypes and testers as fixed factors and location and year as random factors  and . All effects involving *A*_*r*_ are random except (*AT*)_*rv*_ because we do not want to recover inter-year information since there are only two years and the year by tester classification is very disconnected (years do not share testers). Moreover, the (*AT*)_*rv*_ term is analogous to a block factor in an incomplete block design because it is free of *G*_*h*_; therefore, due to the unbalancedness and the small number of years, we can use it as a fixed effect. Furthermore, the main year effect (*A*_*r*_) can be dropped considering that the adjustment of the genotype means is the same for *A*_*r*_+(*AT*)_*rv*_ as for only (*AT*)_*rv*_.

Including all the effects, the final model (4) is


To minimise the loss of information in the transition to the GP stage, we weighted the adjusted means using the inverse of the squared standard errors, which is also appropriate since we are not fitting random block effects [9].

#### Third stage: Genomic prediction

At the third stage, the dataset of *p* markers was merged with the *n* grain dry matter yield adjusted means by years of evaluated models. GP was performed using ridge-regression best linear unbiased prediction (RR-BLUP), where the genotypic values are predicted using the marker information by regressing each SNP on the phenotype [24].

The model was
5

where, *M*^(2)^ is the *n*×1 vector of phenotypic records, here, containing the adjusted means calculated from the second stage, **X** and **β** are, respectively, the design matrix and parameter vector of fixed effects, **Z** is the *n*×*p* marker matrix, whose elements *z*_*hm*_ represent the SNP genotype of the *m*-th marker of the *h*-th genotype entry and take the values -1, 0, or +1 for the aa, Aa, and AA genotypes [24], **u** is the *p*-dimensional vector of SNP effects and **e** is the error vector. The term **Zu** is interpreted as the genetic effect and its estimate  as the GEBV. The GEBV of the *h*-th genotype corresponds to , with *m*=1,⋯,*p* the number of markers,  is the estimated effect of the *m*-th marker and *z*_*hm*_ the SNP genotype of the *m*-th marker for the *h*-th genotype entry. The assumptions of the model are that the error is normally distributed with zero mean and variance **R**[ **e**∼*N*(0,**R**)] and that **u** has a normal distribution with zero mean and variance . **R** is a diagonal matrix with diagonal elements equal to the inverses of the diagonal elements of the inverse of the original variance-covariance matrix of the adjusted means of the second stage [6]. **I**_*p*_ is the *p*-dimensional identity matrix and  represents the proportion of the genetic variance contributed by each individual SNP.

Under the model equation (5) the variance of the observed data is , in which **Γ**=**Z***Z*^*T*^ and **Z**^*T*^ denotes the transpose of **Z** [24]. To speed up the computation, **Γ** was rescaled by replacing **Z** with , with *p* the number of markers [25].

In the year-wise analysis (*Approach 1a*), the genotype adjusted means by year are merged in the *M*^(2)^ vector, and vector *β* contains the intercept and the year effect. In the across-years analysis (*Approach 2*), where year effect was already accounted for, *M*^(2)^ contains the genotype adjusted means and vector *β* contains only the intercept. In the year-wise analysis correcting genotype adjusted means for year effects (*Approach 1b*), the model used did not include a fixed year factor (since we had already adjusted for it) but a common intercept, thus the model was the same as for across-years analysis.

To measure the influence of the relationship among the genotypes on the predictions, we used the adjusted means obtained in the second stage and the pedigree information of the entries in a mixed model testing genotypes and crosses as random effects, so that the variances of both effects would give us an estimation of how much the variation is attributed to the pedigree, e.g. the crosses. The model was
6

where  is the adjusted mean of the *h*-th genotype obtained in the second stage, *G*_*h*_ is the effect of the *h*-th genotype, *C*_*a*_ is the effect of the *a*-th grand parent (gp) cross, e.g. (gp1 × gp2) × (gp3 × gp4), and *e*_*ah*_ the associated error. Additionally, we plotted the relationship heat-map of estimated coefficients of relatedness for individuals based on marker data computed according to Wimmer et al. [26].

#### Cross validation for model comparison

To evaluate model performance, *k*-fold CV was carried out. In CV, the data is split into *k* subsets *t* times. *k*-1 subsets are used as the training set (TS) and the one other subset is the validation set (VS). The TS is used to estimate the parameters that then are used to predict the observations in the VS. The performance of the model was assessed by the Pearson correlation coefficient between the predicted GEBV and the corresponding observations of the VS. This correlation is referred to as predictive ability [23]. As in the first stage, the predictive ability was not adjusted by the square root of the heritability. Although breeding programs are most of the time operating with closely related genotypes, breeders are also interested in knowing the results in a scenario with more distantly related genotypes, for example, using genotypes that share the same grandparents either in the TS or in the VS but not in both. Hence, we wanted to check if accounting for the effect of population structure in the randomisation of CV would make the spatial error models improve the predictive abilities. We chose two scenarios given the relatedness level of the entries and followed the suggested sampling schemes from Albrecht et al. [27], which takes into consideration this fact in the CV procedure. In the first sampling scheme, hereafter called “within crosses” (WC), random sampling is done using all genotypes in the dataset; in the second scheme, hereafter referred to as “across crosses” (AC), genotypes were clustered by cross, so that complete cross-groups were used randomly either in the VS or the TS. There were 349 crosses of different sizes, sharing none, one or two grand parents. The general overview of the methodology is depicted in Figure 2.

### Model selection

**Figure 3:**
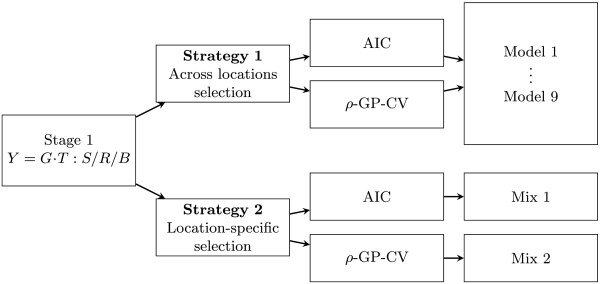
**General representation of strategies to compare model selection methods.** Factors were genotype (*G*), tester (*T*), trial (*S*), replicate (*R*) and block (*B*). Grain dry matter yield (*Y*) is the response variable in the first stage. *Y*=*G*·*T*:*S*/*R*/*B* is the shorthand notation for the model *Y*_*hijkv*_=(*G* *T*)_*hv*_+*S*_*i*_+*R*_*ij*_+*B*_*ijk*_+*e*_*hijkv*_. Datasets of 9 spatial and non spatial models plus one mixed dataset (Mix1) generated from best models given the Akaike information criterion (AIC) and another mixed dataset (Mix2) generated from best models given the predictive abilities (*ρ*-GP-CV).

Two strategies for selecting the best phenotypic model were used in the first stage. In strategy one the best model for all locations is selected, that is, there is no model selection per location but across locations. In strategy two, model selection is location-specific (Figure 3). For both strategies we computed the AIC and performed genomic prediction-cross validation (GP-CV), both per location-year combination. To accomplish the GP-CV approach, we used the adjusted means per location and year of all spatial and non-spatial models. Then, means of genotypes by year-location combination were joined with the molecular marker data to perform GP-CV, in which genetic values were regressed on markers and validation of the model was done using *k*-fold CV. Predictions of unobserved records and predictive abilities of each model were obtained for each year-location combination. We assessed the predictive ability of the models using the Pearson correlation coefficient (*ρ*) between the predicted GEBV and the observed phenotypic value. Hereafter we denote this predictive ability as *ρ*-GP-CV. Predictive abilities were not adjusted with the square root of the heritability, as suggested by Dekkers [28], since this adds an extra error due to heritability computation [15, 23].

For strategy one (across locations model selection), the number of locations with the best fits (either AIC or *ρ*-GP-CV) was counted, so that the model with the best fits in the majority of locations was identified as the best model. For strategy two (location-specific model selection), two datasets were built: “Mix 1”, containing the adjusted means of the locations with the best fit according to the AIC and “Mix 2”, containing the adjusted means of the locations with the highest *ρ*-GP-CV. Thus, after the first stage we had in total eleven data sets of adjusted means, nine corresponding to each tested model from strategy one, plus two more datasets from strategy two: A mixed data set (Mix 1) with the best models per location-year according to the AIC, and another mixed set (Mix 2) with best models per location-year according to the *ρ*-GP-CV.

### Softwares

All analyses were performed using SAS. Stage 1 and 3 used the MIXED procedure and Stage 2 used PROC HPMIXED. Relationship matrix was calculated using the Synbreed Package [29] for R 2.15.

## Results

### First stage - strategy 1: Model selection across locations

In the first stage - strategy 1, we did model selection across locations using AIC and predictive abilities (*ρ*-GP-CV) per location-year combination. According to the AIC, the results favoured the two-dimensional models (Table 5). To do a fair comparison between selection methods using AIC and *ρ*-GP-CV, we first describe AIC for years 2009 and 2010, for which *ρ*-GP-CV were also available and then, as additional information, for year 2012, for which *ρ*-GP-CV was not available since the marker information was missing.

**Table 5:** **Akaike information criterion (AIC) of models at first stage (M1, ⋯, M9) by year and location (L) for grain dry matter yield (Y)**

Year	L	M1	M2	M3	M4	M5	M6	M7	M8	M9
2009	G-L1	101.7	84.3	45.5	47.2	20.4	6.9	45.6	**0**	1.7
2009	G-L2	83.1	67.5	50.9	38.5	31.4	20.7	40.7	0.5	**0**
2009	G-L3	45.7	30.4	41.5	31.1	40.1	26.9	31.2	1.0	**0**
2009	G-L4	125.0	19.1	125.1	114.9	90.3	19.6	115.5	65.0	**0**
2009	G-L5	29.1	8.0	18.1	24.5	15.3	1.2	–	12.3	**0**
2009	G-L6	51.6	47.6	37.7	29.5	41.7	35.4	29.4	**0**	1.2
2009	G-L7	81.5	56.1	55.3	62.8	36.5	11.0	55.5	5.1	**0**
2009	P-L1	126.4	115.6	121.6	116.3	109.5	108.8	116.2	**0**	1.9
2009	P-L2	62.3	45.4	62.4	54.6	57.3	47.2	54.9	1.5	**0**
2009	P-L3	120.9	65.9	116.1	105.5	99.7	49.6	105.5	17.3	**0**
2009	P-L4	145.9	98.6	132.8	126.4	126.4	80.1	126.4	0.4	**0**
2010	G-L1	35.5	4.9	35.6	31.5	12.3	**0**	32.0	12.3	1.8
2010	G-L2	25.0	7.2	27.0	21.7	29.7	11.9	19.7	**0**	*-3.2*
2010	G-L4	141.4	74.2	128.7	117.1	130.2	57.4	118.4	5.0	**0**
2010	G-L5	21.6	**0**	23.4	22.9	21.9	3.3	22.9	22.1	2.8
2010	G-L6	80.9	60.0	72.8	59.8	55.4	41.5	61.1	**0**	0.6
2010	G-L7	69.5	22.3	56.2	47.8	37.2	23.6	48.1	2.6	**0**
2010	G-L8	40.8	24.7	32.1	22.6	27.7	19.6	23.1	**0**	1.4
2010	P-L1	38.8	5.7	38.8	38.8	39.4	9.4	40.8	39.1	**0**
2010	P-L2	40.0	0.7	41.6	36.1	39.8	4.1	36.9	4.3	**0**
2010	P-L3	66.4	**0**	68.4	67.2	69.5	3.7	70.4	71.5	5.7
2010	P-L4	95.0	80.4	90.5	79.1	87.0	66.7	79.4	**0**	3.2
Counts		0	2	0	0	0	1	0	7	**12**
		0%	9%	0%	0%	0%	5%	0.00	32%	**55%**
2012	G-L4	35.3	**0**	35.3	36.2	26.0	0.6	35.3	24.2	–
2012	G-L5	66.3	2.6	67.0	66.3	42.1	5.9	–	21.5	**0**
2012	G-L6	148.4	131.4	93.8	93.7	18.7	18.7	89.9	**0**	**0**
2012	G-L7	38.3	4.5	40.3	38.3	36.3	**0**	42.3	–	1.9
2012	G-L8	45.3	39.8	37.7	33.5	35.6	37.3	33.9	1.9	**0**
2012	G-L9	402.3	321.5	200.9	181.7	81.9	81.9	191.6	**0**	**0**
2012	G-L10	39.7	**0**	41.5	41.4	22.1	3.5	43.5	6.7	1.1
2012	G-L11	18.0	**0**	19.7	18.0	8.4	1.2	21.6	3.7	–
2012	P-L1	189.5	168.8	158.9	148.9	146.3	137.8	149.1	**0**	1.7
2012	P-L2	127.4	49.3	129.1	122.6	129.7	49.9	123.9	5.9	**0**
2012	P-L3	107.8	55.3	103.1	95.0	101.0	49.3	96.1	7.9	**0**
2012	P-L4	226.3	0.2	226.3	222.1	226.3	**0**	226.3	226.3	2.0
2012	P-L5	13.2	**0**	13.2	13.2	11.9	1.5	13.2	13.9	3.5
2012	P-L6	79.0	54.8	70.4	66.9	65.8	37.9	67.0	**0**	1.7
Counts		0	4	0	0	0	2	0	4	**6**
		0%	29%	0%	0%	0%	14%	0%	29%	**43%**

Table shows *Δ*AIC relative to the best model. Boldfaced entries in the table indicate best model (fit) within location. Empty cells (–) correspond to locations where the model did not converge. In italics, we report the models that converged but the Hessian matrix was not positive definite.

For years 2009 and 2010, M9 and M8 had the majority of best fits across locations. M9 (Baseline + row + column and AR(1) × AR(1) + nugget) resulted in 12 out of 22 cases as the best model. M8 (Baseline and AR(1) × AR(1) + nugget) was best in 7 out of 22 cases. The baseline model + row + column (M2) fitted the best 9% of the times and M6 5% of the times.

A similar tendency was observed in 2012, where 43% of the times (6 out of 14) M9 had the best fit and M8 was best 29% of the times. For this year 2012, models M7, M8 and M9 could not be fitted in some locations. Another third of the times (29%), M2 had best fits. Interestingly, M2 had the best fits in the locations that had convergence problems for models M8 and M9. M1, M3, M4, M5 and M7 never had best fits in any of both groups of years.

The predictive abilities (*ρ*-GP-CV) per location-year combination showed a rather different pattern for best models within locations; however, the two-dimensional models were also more frequently selected than one-dimensional models (Table 6). M8 (Baseline and AR(1) × AR(1) + nugget) showed in seven of 22 settings the highest *ρ*-GP-CV per location-year combination followed by M9 (Baseline + row + column and AR(1) × AR(1) + nugget) with six out of 22 times. The baseline model + row + column (M2) was selected twice and models M3, M4 and M6 had also one, three and three selections out of 22, respectively. M1, M5 and M7 had no best fits at all.

**Table 6:** **Predictive abilities of observed and predicted values of a** ***5*** **-fold-CV by year-location combination of models at first stage (M1,** ***⋯*** **, M9) for grain dry matter yield (Y), and repeatability (** ***R*** **) of the trait by location**

Year	Loc	M1	M2	M3	M4	M5	M6	M7	M8	M9	***R***
2009	G-L1	0.469	0.473	0.462	0.481	0.448	0.455	0.474	**0.481**	0.478	0.376
2009	G-L2	0.271	0.272	0.279	0.280	0.282	**0.288**	0.282	0.270	0.269	0.177
2009	G-L3	0.347	0.344	0.351	0.350	0.345	0.339	0.350	**0.355** ^*§*^	0.355	0.264
2009	G-L4	0.595	0.593	0.597	**0.602** ^*§*^	0.592	0.594	0.602	0.592	0.598	0.440
2009	G-L5	0.495	0.514	0.506	0.505	0.519	0.527	–	0.514	**0.529**	0.303
2009	G-L6	0.393	**0.398**	0.357	0.372	0.359	0.360	0.369	0.372	0.378	0.077
2009	G-L7	0.596	0.594	0.586	**0.599**	0.578	0.565	0.591	0.584	0.577	0.299
2009	P-L1	0.127	0.118	0.132	0.138	0.116	0.114	0.138	**0.174**	0.167	0.225
2009	P-L2	0.301	0.306	0.303	0.310	0.307	0.309	0.310	0.323	**0.323** ^*§*^	0.338
2009	P-L3	-0.154	-0.165	-0.153	-0.154	-0.169	-0.172	-0.154	-0.158	-0.175	0.247
2009	P-L4	0.520	0.518	0.527	0.525	0.520	0.522	0.525	**0.558**	0.555	0.362
2010	G-L1	0.428	0.471	0.426	0.432	0.464	**0.478**	0.431	0.466	0.475	0.263
2010	G-L2	0.394	0.392	0.399	**0.407**	0.400	0.398	0.406	0.401	*0.400*	0.248
2010	G-L4	0.470	0.472	**0.477** ^*§*^	0.476	0.478	0.477	0.477	0.404	0.424	0.326
2010	G-L5	0.469	0.485	0.471	0.469	0.476	0.486	0.469	0.479	**0.487**	0.407
2010	G-L6	0.576	0.583	0.601	0.612	0.601	0.608	0.611	**0.619**	0.618	0.310
2010	G-L7	0.520	0.552	0.557	0.564	0.541	0.556	0.565	**0.579**	0.574	0.298
2010	G-L8	0.589	0.600	0.599	0.597	0.605	0.605	0.598	0.603	**0.607**	0.540
2010	P-L1	0.327	0.334	0.327	0.327	0.326	0.333	0.327	0.327	**0.337**	0.439
2010	P-L2	0.277	0.310	0.275	0.266	0.275	0.309	0.268	**0.311**	0.307	0.436
2010	P-L3	0.461	0.466	0.461	0.462	0.459	**0.467**	0.461	0.459	**0.467**	0.416
2010	P-L4	0.314	**0.322**	0.317	0.316	0.315	0.317	0.317	0.317	0.315	0.360
Counts		0	2	1	3	0	3	0	**7**	**6**	
		0%	9%	5%	14%	0%	14%	0%	32%	27%	

Boldfaced entries in the table indicate best model (fit) within location. Empty cells correspond to locations where the model did not converge. In italics, we report the models that converged but the Hessian matrix was not positive definite. *§* Better than second best model at forth decimal place

One location of 2009 (P-L3) produced a negative predictive ability for all models. We did not consider this location in the counting of best fits, since a higher negative number is actually a worse fit in regard to predictions, but low or high negative are both interpreted as zero prediction. Despite the negative correlations, this location was included in the mixed datasets produced from the site-specific model selection. We used the adjusted means produced from the baseline model. Another location (G-L1 2009) showed way lower predictive abilities than the rest of the locations. To understand these two situations, we calculated the repeatability of the trait in each location for the baseline model. The repeatability *R* is defined as the ratio of the between-individual component to the total phenotypic variance [30], which in our case, and following the methodology described by Nakagawa and Schielzeth [31], corresponds to
7

where  is the between-groups variance and corresponds to the variance of the effect (*GT*)_*hv*_ fitted as random effect, and in the denominator, the total phenotypic variance given by the sum of the between-groups variance  and the within-groups variances, i.e. replicates within trials  and blocks within replicates  plus the residual variance . The interpretation of this repeatability strictly refers to the expected within-group correlations among measurements, i.e. the agreement among measurements; thus, the gist of the definition of repeatability is related to the reproducibility of the absolute values of measurements. A slightly higher repeatability in Cycle1-2009 was observed for location G-L4 (Table 6), which involved more trials, i.e. more genotypes, in comparison with other locations in Germany. The trend in Cycle1-2010 was in favour of the Polish locations, which overal l had more homogeneous and higher repeatabilities. We discuss the relation between repeatabilities and predictive abilities in the next section.

### Second stage: fitting genotypes by year vs. across years

From a methodological point of view, fitting the year effect in the GP stage was easier and more direct than accounting for the year effect in the second stage, in the sense that the model for the latter approach became too complex and the variance covariance matrix of adjusted means was not possible to be produced using the procedure HPMIXED of SAS given the high computer power required. Instead, we computed the adjusted means with corresponding standard errors, which were then used to do the weighting to pass on from the second to the third stage.

**Figure 4:**
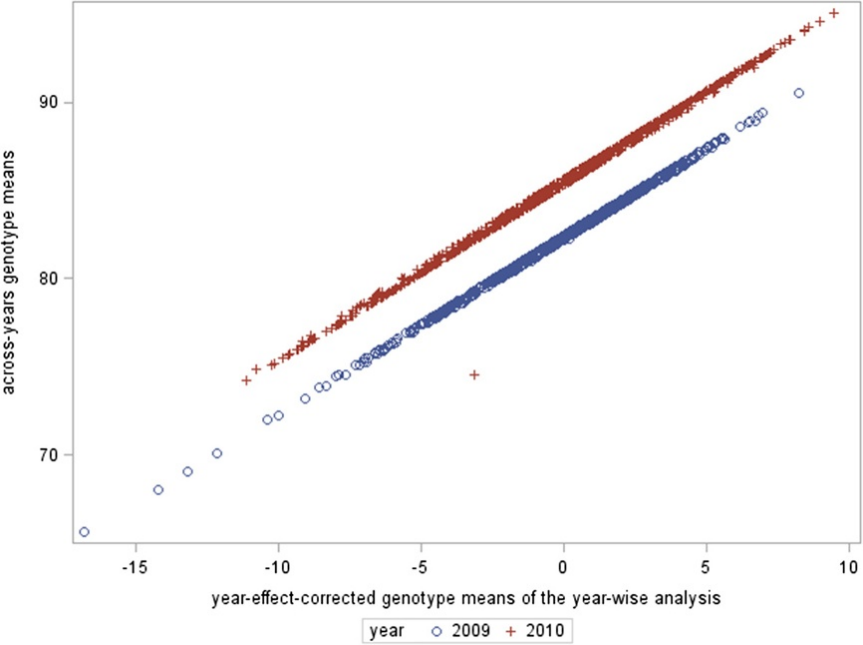
**Comparison of approaches for year adjustment.** In the x-axis, the genotype adjusted means across-year analysis are plotted. In the y-axis, the year-effect-corrected adjusted means from the year-wise analysis are depicted.

The adjusted means obtained from the across-years analysis (*Approach 2*) were plotted against the year effect-corrected genotype adjusted means (from *Approach 1b*) to compare the difference of adjustments, in the former case based on one single check against the adjustment given the simple mean of the genotypes in each year (Figure 4). Below the two principal lines, an observation corresponding to the shared check across years stood out from the others, reflecting the year adjustment. At first glance, it is clear that the check was the only observation pulled down implying that the year adjustment of this check was not strong enough to pull down the observations of the whole year. Both approaches were examined later using the predictive abilities obtained in the GP stage.

### Third stage: genomic prediction

The predictive abilities of the GP stage were taken as the definitive decision criterion for identifying the best strategy for model selection, the best model, and the most reliable approach to account for year effects, and to identify the consequences of population stratification in GP. We start by presenting results of the comparison of the approaches used for fitting the year effect, since with these we only used the baseline model. Then we present the differences between sampling methods for CV together with the comparison of the models and the model selection strategies.

#### Comparison of approaches to account for year effect in GP

**Figure 5:**
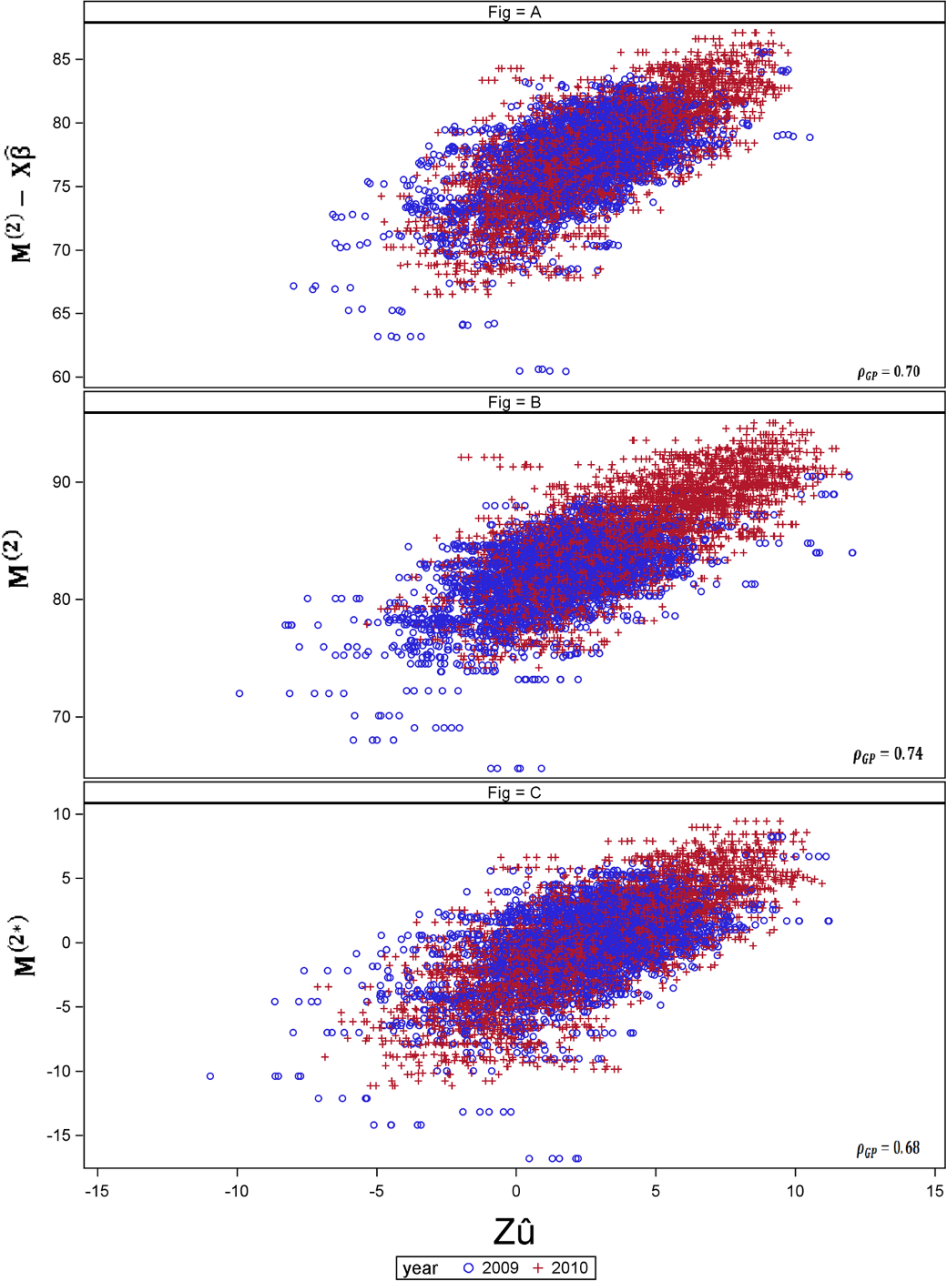
**Comparison between approaches to fit the year effect.** The y-axis represents the genotype adjusted means  in **(A)**, *M* ^(2)^ in **(B)** and *M* ^(2∗)^ in **(C)** ] and the x-axis represents the GEBV (). (A) Year-wise analysis (*Approach 1a*), fitting year as fixed effect in the GP stage, (B) Across-years analysis (*Approach 2*), using year in the second stage and (C) year-wise analysis using the year effect-corrected genotype means (*Approach 1b*). *ρ*_*GP*_ represents the predictive ability.

The GP-CV for the approach using the year as a fixed term in the third stage (*Approach 1a*) yielded a predictive ability of 0.70 (Table 7), whereas predictive ability for the approach accounting for a fixed year effect in the second stage (*Approach 2*) was 0.74. The predictive ability reached 0.68, using the year-effect-corrected adjusted means in the GP-CV (*Approach 1b*). The scatter plots of GEBV () against the observed phenotypic values (adjusted means) in the three cases are depicted in Figure 5. In *Approach 1a*, we plotted the GEBV against the corrected observed phenotypic values, calculated as , where *M*^(2)^ is the vector of genotype adjusted means obtained in the second stage and  the predicted year effect (Figure 5A). For *Approach 2*, the observed phenotypic values *M*^(2)^ against  are shown (Figure 5B). For *Approach 1b*, *M*^(2∗)^ against  are plotted, with *M*^(2∗)^ the year-effect-corrected adjusted means of genotypes (Figure 5C).

**Table 7:** **Predictive abilities between observed and predicted values for 9 spatial and non-spatial models (M1,** ***⋯*** **, M9) and mixed datasets using the best locations given the AIC (Mix1) and the** ***ρ*** **-GP-CV per location-year combination (Mix2)**

	M1	M2	M3	M4	M5	M6	M7	M8	M9	Mix1	Mix 2
WC	0.700	0.694	0.691	0.679	0.692	0.692	0.691	0.694	0.689	0.689	0.690
	a	ab	ab	c	ab	ab	ab	ab	abc	bc	abc
AC	0.395	0.398	0.390	0.395	0.391	0.389	0.389	0.395	0.391	0.391	0.390
	b	a	cd	de	c	e	de	b	c	c	cd

Same letters within rows indicate no significant differences (*α*=5*%*) according to a paired t-test. Sampling strategies were: Within crosses (WC) and across crosses (AC).

#### Comparison of model selection strategies using different sampling methods in cross validation

Fitting model (6) to measure the influence of the relationship among genotypes on predictions yielded variance components for genotypes, crosses and error for year 2009 of 4.03, 3.67 and 1.66, respectively, and for year 2010 of 4.72, 10.70 and 1.32, respectively. Thus, the cross effect in 2009 is contributing in about 40*%* and in the next year more than 60*%* to the total variation explained by the data.

**Figure 6:**
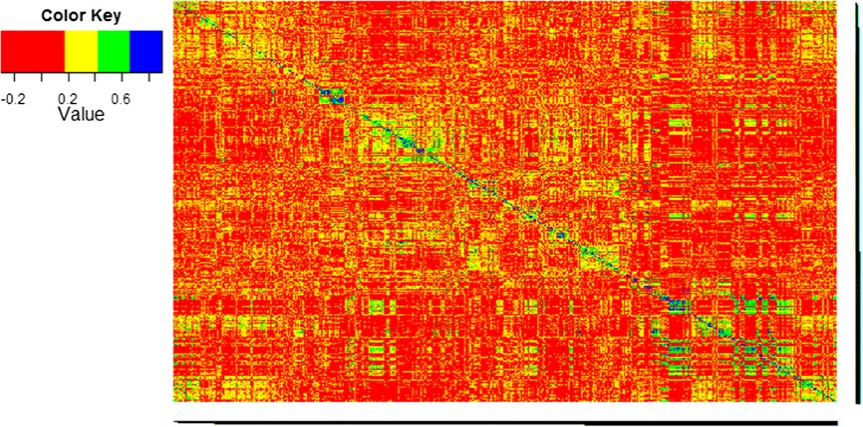
**Marker-based relationship heat-map.** Visualised are pairwise relationship coefficients estimated from the maker data for genotypes of years 2009 and 2010. Higher values represent a stronger relationship.

The marker-based relationship heat-map (Figure 6) shows some clusters among genotypes of the same cross indicating genetic relatedness. The predictive abilities using five times 5-fold CV of datasets resulting from first stage analysis of all spatial and non-spatial models plus the mixed datasets were in general very similar within sampling strategies (Table 7). For the across-crosses (AC) sampling scheme, the predictive abilities were lower than the ones obtained with the within-crosses (WC) sampling scheme. In the AC sampling, we fixed the initial seed of the random number generator used for randomization in the CV procedure at the same value for all models to be able to compare the models when the same crosses were used in the training set.

We compared the models and the sampling methods using a paired t-test (*α*=5*%*) by resembling a randomized complete block design, where the predictive ability of each repetition of the CV was taken as a block, thus accounting for the dependence among observations from the same samples (Table 7). For the first sampling method (WC), three groups were identified with some overlaps, but showing not much of a difference among models. From the across-crosses sampling strategy (AC), five groups were distinguished with some overlaps: M2 had the highest predictive ability and models M4, M6 and M7 had the worst predictive abilities.

Potential bias of GP is another important element that could be used to compare models. We computed the bias as suggested by [32, 33]. The comparison of the biases of all models followed a rather similar trend as the predictive abilities showed in Table 7. We present the analysis of bias as supplementary material (Additional file 2).

The heritability (square root of heritability) for the baseline model was estimated as 0.68 (0.82) for year 2009, 0.73 (0.85) for year 2010 and 0.69 (0.83) for 2012 using the equation (3). In principle, the *ad hoc* method may approximate the true value of heritability but making the unrealistic assumption of uncorrelated genotypes [23]. We computed the heritability to have a rough idea of how much could we expect from the predictive abilities. The predictive ability divided by square root of heritability is an estimate of the accuracy of GP [23], and the square root of the heritability provides the upper bound for the predictive ability [30], thus one expects that the predictive abilities are not very far from the square root of heritability. In this case, the square roots of the heritabilities are somewhat larger than the corresponding predictive abilities, indicating that the predictions are not sufficiently accurate due to limited data size, thus not exhausting completely the genetic variance. To explore in which extend could have our models explained the variance not captured by the markers, we fitted an additional component accounting for the polygenic effect in the GP stage [24]. The baseline model (M1) yielded a genotypic variance of 2.99; when we incorporated the polygenic effect, the genotypic variance was 2.72 and polygenic variance was 0.36, indicating that about 88% of the total genetic variance was captured by the RR-BLUP model.

## Discussion

Selecting the models at the first stage produced different results than assessing them in the third stage. AIC had better scores for the models that used row and column effects, e.g. Models M9, M6 and M2 (Table 5) or M8 that had a 2-dimensional variance-covariance error structure. *ρ*-GP-CV also picked M8 and M9 (Table 6) but the choices were more spread over the models covering even the baseline model. In general, in the first stage, both AIC and *ρ*-GP-CV produced better scores for the two-dimensional models, whereas in the third stage the baseline and one-dimensional models seemed to be better than the more complex models (Table 7). The explanation of this pattern may be related to the second stage, where the interaction genotype × location played a role. The two-dimensional models performed very well in modelling heterogeneity within field, but when the means were integrated across the whole experiment, including all locations and years, the two-dimensional spatial error models seemed to over-adjust the means, yielding a poorer predictive ability in the GP stage. The one-dimensional spatial error models and the two-dimensional model without spatial error structure were sufficient to estimate appropriately adjusted means. This corroborates Piepho and Williams [21] who concluded that for small portions of a field, a particular spatial model may hold well but if fitted all across the field it may fail. In a wheat experiment, Lado et al. [34] found that using moving averages as covariable significantly improved the predictive abilities of GP. They recognised strong heterogeneous patterns of irrigation in the field, that were not controlled with a single blocking system.

Models M1, M3 and M7 were never selected as having the best fits either by AIC or *ρ*-GP-CV. These models had in common that none of them used rows and columns as additional factors, strengthening the conclusion that row-column designs may have the potential to correctly control field heterogeneity and thus enhance predictive ability of genomic prediction.

Fitting a location-specific error model did not have an advantage over fitting a common model across locations. Neither did the dataset composed of means computed using models have best AIC fits (Mix 1) nor the second dataset containing the means computed using models with highest *ρ*-GP-CV (Mix 2) produce better predictive abilities in the GP stage.

The models with nugget had better fits than the corresponding baseline model without the nugget. The drawback was that fitting those models was not straightforward, since almost every location required a separate coding specifying initial values and lower boundary constraints on the covariance parameters. Good statistical and biological reasons have been presented of why including a nugget to analysis of field experiment is beneficial [35].

If we ignore the two-dimensional spatial models (M5, M6, M8 and M9), the AIC privileges M2 and *ρ*-GP-CV yields more diverse results with the majority of choices for M2 and M4. In fact, when the spatial component of a resolvable row-column design based on linear variance (LV) does not lead to an improved fit, returning to classical row-column design provides randomisation protection [36].

Williams and Luckett [37] performed studies aiming to find the optimal plot size, the optimal plot arrangements and the best spatial model (the so-called uniformity trials) and showed that in cotton and barley row and column designs are well suited for variety testing in plant breeding trials. Moreover, recent simulation studies from Möhring et al. [38] showed that designs including rows and columns outperformed one-dimensional blocking. In the same work, the authors mention that blocking in the direction of plots with common long sides is preferable, which is common in cereal breeding [39].

We cannot affirm that *ρ*-GP-CV was better than AIC for model selection or vice versa, nor that the results showed the same trend; but if we would have used either of these two strategies to select the best model, we would have selected the M9 with AIC or M8 with *ρ*-GP-CV. The GP predictive ability obtained by M2 (Table 7) was slightly better than M8 and M9 (specifically AC sampling method); however, this model (M2) was not highlighted by either of the two selection criteria (AIC or *ρ*-GP-CV).

In practice, the fact that there were no large statistical differences is good news for the breeders because the baseline model (M1), or even better, the simplest model with row-column adjustment (M2), are appropriate for phenotypic analysis towards GP.

As a model selection method, GP-CV is of interest because it may allow to compare models with different fixed effects, even when REML is used for estimating the variance parameters. No simple recommendation has been reported concerning the best model selection criterion in the case of spatial models [13, 40]. Predictive abilities have been used between environments as similarity measure and then to join similar environments into clusters [15]. Thus, in a sense *ρ*-GP-CV allows giving an interpretation to the environment under scrutiny and the displayed trend do not depart far from the classical AIC. The repeatabilities (*R*) presented in parallel to the *ρ*-GP-CV (Table 6) show a low correlation (*ρ*=0.36, p-value = 0.0965) with the predictive abilities from the baseline model. In fact, we expected that for location P-L3 of 2009, which had a negative predictability, the *R* was very low almost zero, but this was not the case; hence we could not conclude that the low predictive ability is mainly due to environmental effects. Riedelsheimer et al. [41] also reported negative predictive accuracies when testing unrelated crosses in the CV procedure and observed that using unrelated crosses could have provided a negative prediction signal due to opposite linkage phases with important QTL displayed in the TS, suggesting that the negative predictive accuracies are associated with the marker pattern.

In this study we explored three ways to adjust the year effect given the weak connectivity across years. Using the single check (*Approach 2*) to make the year adjustment was not a better choice than adjusting by the simple year mean (*Approach 1b*) or accounting for the year effect in the GP stage (*Approach 1a*), even though the estimated predictive ability was the highest. The “year clouds” produced using *Approach 2* (Figure 5B) did not overlap perfectly, from which we concluded that the correction was not appropriate and generated an over-fitting of the markers in the GP-CV procedure due to the fact that markers also predicted the year effect and not the SNP-effects alone. Using the year-mean correction for adjusted means in the second stage (*Approach 1b*) produced a lower *ρ*-GP-CV, that, given the overlay of the clouds of predicted vs. observed values, seems to be more realistic. However, fitting the year effect manually, i.e. using ordinary least squares estimation (OLSE) vs. fitting it as a fixed effect in the GP stage, i.e. using generalised least squares estimation (GLSE) can definitively yield a more precise estimate. Indeed, the residual variance in *Approach 1b* using year effect-corrected adjusted means was around 3.9 (in average for the five replicates) and in *Approach 1a* using the year fixed effect in the GP stage yielded residual variance of 3.0 (in average for the five replicates). In *Approach 1a*, where we fitted the year in the GP stage, we removed the year effect from the observed adjusted means derived from the second stage  to avoid bias of the predictive abilities; however, there would still be some bias because the subtracted year effect was not the true effect but an estimate of the year effect.

Models were eventually assessed and compared using the *ρ*-GP-CV in the third stage. The two sampling scenarios to perform the CV procedure aimed to recreate the cases where the material was genetically close, with some individuals coming from the same parental cross, and more distantly related to avoid individuals from the same parental cross in the randomisation procedure of CV. This more distantly related material shows some identical-by-state (IBS) similarity, therefore it was not unrelated in the theoretical sense of population genetics. This more distantly related scenario may be seen also as a case where one tries to predict a scenario whose linking information is weak or lacking, e.g. different genotypes and/or locations in the TS and VS [42–44].

The predictive abilities obtained for GP using WC sampling were located in the middle-high range and using AC sampling, predictive abilities were placed in the middle range. The predictive ability of the AC sampling was significantly lower than WC, as expected for GP of a dataset showing population structure. Riedelsheimer et al. [41] drew similar conclusions using unrelated biparental maize families. They concluded that predictive accuracy could be increased by adding crosses (families) sharing both parents to the TS. In this respect, the use of pedigree and marker information to borrow information from both sources is suggested [44].

## Conclusions

The main conclusions of this study are: (i) Fitting a traditional model including row and column factors across all locations was good enough to account for field heterogeneity in the first stage under GP frame. This also suggests that row-column designs may be preferable to designs with a single blocking factor; (ii) AIC and *ρ*-GP-CV did not have the same trend in selecting across models, but both favoured in the end models M8 and M9; however, none of the methods picked the model with highest predictive ability. Fitting a location-specific error model did not produce an advantage over fitting a common model across locations; (iii) the baseline model (M1) and the simplest row-column adjustment (M2) had in overall the best results, which is very good news since in routine analysis complex models may require much programming expertise and powerful computers; (iv) in a dataset weakly connected across years, a more reasonable model-wise structure is to account for the year factor in the genomic prediction stage rather than in a previous stage, to ensure that the effect is not confounded with the markers adjustment, and (v) datasets of distantly related genotypes may have a poor performance for GP purposes; however, increasing the size of the crosses may be an opportunity to enhance predictive ability in these cases of disconnected datasets on related sets of genotypes.

## Electronic supplementary material

Additional file 1: **SAS codes (version 9.3) used to implement first stage of phenotypic analysis referred in Table** 4 **.** (PDF 40 KB)

Additional file 2: **Analysis of bias of genomic prediction.** (PDF 54 KB)

## Abbreviations

GP: Genomic prediction
GEBV: Genomic estimated breeding value
SNP: Single nucleotide polymorphism
MET: Multi-environment trial
CV: Cross validation
AIC: Akaike information criterion
BIC: Bayesian information criterion
REML: Restricted maximum likelihood
ML: Maximum likelihood
MAF: Minor allele frequency
*ρ*-GP-CV: Predictive ability
BLUE: Best linear unbiased estimator
RR-BLUP: Ridge regression-best linear unbiased predictor
VS: Validation set
TS: Training set
WC: Within-crosses
AC: Across-crosses
OLSE: Ordinary least squares estimation
GLSE: Generalised least squares estimation
IBS: Identical-by-state.
