# Supplementary material for: The importance of phenotypic data analysis for genomic prediction - a case study comparing different spatial models in rye
Source: BMC Genomics. 2014 Aug 4;15(1):646. doi: 10.1186/1471-2164-15-646 (PMC4133075; doi:10.1186/1471-2164-15-646)
Supplement: Supplementary file 1 — Additional file 1: SAS codes (version 9.3) used to implement first stage of phenotypic analysis referred in Table 4 . (PDF 40 KB) [file 12864_2014_6343_MOESM1_ESM.pdf]

Additional file 1. SAS codes (version 9.3) used to implement first stage of phenotypic analysis referred in Table 4.

```
/********************************************************************
*********************** Appendix A*************************************
********** Some SAS code to fit the models of the first stage *********
*************************referred in Table 4***************************
*********************************************************************/

**** Model 1. Baseline model ------------------------------;

proc mixed data=data1 ;
ods output FitStatistics=fits_M1 lsmeans= adjmeans_M1 covparms=cp_M1;
by year loc;
class year loc genotype tester trial rep block  row column ;
model y1=genotype*tester/ddfm=residual solution ;
random int rep rep*block / sub=trial;
lsmeans genotype*tester /cov;
run;


**** Model 2. Baseline + row + column -----------;

proc mixed data=data1;
ods output FitStatistics=fits_M2 lsmeans= adjmeans_M2 covparms=cp_M2;
by year loc;
class year loc genotype tester trial rep block row column ;
model y1=genotype*tester/ddfm=residual solution ;
random int rep rep*block / sub=trial;
random row / sub=trial*rep;
random column /sub=trial*rep;
lsmeans genotype*tester /cov;
run;


**** Model 3. Baseline --------- AR(1) ;

proc mixed data=data1;
ods output FitStatistics=fits_M3 lsmeans= adjmeans_M3 covparms=cp_M3;
by year loc;
class year loc genotype tester trial rep block row column plot ;
model y1=genotype*tester/ddfm=residual solution ;
random int rep rep*block / sub=trial;
repeated plot / sub=trial*rep*block type=AR(1);
lsmeans genotype*tester /cov;
parms 1 1 1 0.1 1 / lowerb=  . , . , . , 1e-8 , . ;
run;


*** Model 4. Baseline --------- LV + nugget ;

proc mixed data=data1;
ods output FitStatistics=fits_M4 lsmeans= adjmeans_M4 covparms=cp_M4;
by year loc;
class year loc genotype tester trial rep block row column plot ;
model y1=genotype*tester/ddfm=residual solution ;
random int rep rep*block / sub=trial;
random plot / sub=trial*rep*block type=LIN(1)  ldata=LV_matrix;
lsmeans genotype*tester /cov;
parms 1 1 1 0.1 1 / lowerb=. , . , . , 1e-8, . ;
```

```
run;


*** Model 5. Baseline --------- AR(1) x AR(1);

proc mixed data=data1;
ods output FitStatistics=fits_M5 lsmeans= adjmeans_M5 covparms=cp_M5;
by year loc;
class year loc genotype tester trial rep block row column plot ;
model y1=genotype*tester/ddfm=residual solution ;
random int rep rep*block / sub=trial;
repeated row*column / sub=trial*rep type=SP(POWA) (ro co);
lsmeans genotype*tester /cov;
parms 1 1 1 0.1 0.1 1 / lowerb=  . , . , . , 1e-8 , 1e-8 , . ;
run;


*** Model 6. Baseline + row + column --------- AR(1) x AR(1);

proc mixed data=data1;
ods output FitStatistics=fits_M6 lsmeans= adjmeans_M6 covparms=cp_M6;
by year loc;
class year loc genotype tester trial rep block row column plot ;
model y1=genotype*tester/ddfm=residual solution ;
random int rep rep*block / sub=trial;
random row / sub=trial*rep;
random column/ sub=trial*rep;
repeated row*column / sub=trial*rep type=SP(POWA) (ro co); *ro and co are
numerical variables with row and column coordinates;
lsmeans genotype*tester /cov;
parms 1 1 1 1 1 0.1 0.1 1 / lowerb= . , . , . , . , . , 1e-8 , 1e-8 , . ;
run;


*** Model 7. Baseline  --------- AR(1) + nugget ;

proc mixed data=data1;
ods output FitStatistics=fits_M7 lsmeans= adjmeans_M7 covparms=cp_M7;
by year loc;
class year loc genotype tester trial rep block row column plot ;
model y1=genotype*tester/ddfm=residual solution ;
random int rep rep*block / sub=trial;
repeated plot / sub=trial*rep*block local type=AR(1);
lsmeans genotype*tester /cov;
parms (0 1) (1) (1) (1) (0.1) (1) / lowerb= . , . , . , . , 1e-8 , . ;
run;


*** Model 8. Baseline  --------- AR(1) x AR(1) + nugget;

proc mixed data=data1;
ods output FitStatistics=fits_M8 lsmeans= adjmeans_M8 covparms=cp_M8;
by year loc;
class year loc genotype tester trial rep block row column plot ;
model y1=genotype*tester/ddfm=residual solution ;
random int rep rep*block / sub=trial;
random row / sub=trial*rep;
repeated row*column / sub=trial*rep local type=SP(POWA) (ro co); *ro and co
are numerical variables with row and column coordinates;
lsmeans genotype*tester /cov;
parms 1 1 1 1 0.1 0.1 1 / lowerb=  . , . , . , . , 1e-8 , 1e-8 , . ;
```

```
run;


*** Model 9. Baseline + row + column --------- AR(1) x AR(1) + nugget;

proc mixed data=data1;
ods output FitStatistics=fits_M8 lsmeans= adjmeans_M8 covparms=cp_M8;
by year loc;
class year loc genotype tester trial rep block row column plot ;
model y1=genotype*tester/ddfm=residual solution ;
random int rep rep*block / sub=trial;
random row / sub=trial*rep;
random column/ sub=trial*rep;
repeated row*column / sub=trial*rep local type=SP(POWA) (ro co); *ro and co
are numerical variables with row and column coordinates;
lsmeans genotype*tester /cov;
parms 1 1 1 1 1 1 0.1 0.1 1 / lowerb= . , . , . , . , . , 1e-8 , 1e-8 , .
;*hold=6,7;
run;
```
