# Supplementary material for: The importance of phenotypic data analysis for genomic prediction - a case study comparing different spatial models in rye
Source: BMC Genomics. 2014 Aug 4;15(1):646. doi: 10.1186/1471-2164-15-646 (PMC4133075; doi:10.1186/1471-2164-15-646)
Supplement: Supplementary file 2 — Additional file 2: Analysis of bias of genomic prediction. (PDF 54 KB) [file 12864_2014_6343_MOESM2_ESM.pdf]

Additional file 2. Analysis of bias of genomic prediction.

One reviewer pointed out that genomic prediction is biased and suggested to investigate the potential bias by regressing observations on the predictions from the same cross validation procedure. Other authors (Le Roy et al., 2012; Wang et al., 2012) have used the method to compare genomic prediction models that make use of different penalization tools, such as RR-BLUP, Bayes, Lasso or any other machine learning method. Although we use here only RR-BLUP, we computed the bias of each error spatial model.

Results

Below the results for bias of the models and the mixed datasets for both sampling strategies (WC and AC).

Table: *Bias (regression coefficient between observations and predictions) for 9 spatial and non-spatial models (M1, …, M9) and mixed datasets using the best locations given AIC (Mix1) and ρ-GP-CV (Mix2). Comparisons were performed using the absolute deviation of the regression coefficient from one. Same letters within rows indicate no significant differences (α = 5%) according to a paired t-test. Sampling strategies were: Within crosses (WC) and across crosses (AC).*

	M1	M2	M3	M4	M5	M6	M7	M8	M9	Mix1	Mix2
WC	0.958	0.959	0.944	0.916	0.940	0.939	0.947	0.950	0.933	0.933	1.138
	ab	a	ab	c	abc	abc	ab	ab	bc	bc	d
AC	0.553	0.556	0.546	0.545	0.551	0.549	0.544	0.553	0.548	0.546	0.539
	ab	a	def	ef	bc	cd	f	bc	de	def	g

For both strategies, it turned out that the less biased model was M2, confirming the conclusions throughout the paper that the model with the simplest row-column adjustment had in overall the best results.
